# Supplementary material for: Maternal NAT10 orchestrates oocyte meiotic cell-cycle progression and maturation in mice
Source: Nat Commun. 2023 Jun 22;14:3729. doi: 10.1038/s41467-023-39256-0 (PMC10287700; doi:10.1038/s41467-023-39256-0)
Supplement: Supplementary file 1 — Supplementary Information [file 41467_2023_39256_MOESM1_ESM.pdf]

Supplementary Materials

Maternal NAT10 orchestrates oocyte meiotic cell-cycle progression and maturation in mice

Xue Jiang[1,†], Yu Cheng[2,†], Yuzhang Zhu[3,†], Caoling Xu[1,†], Qiaodan Li[4,], Xuemei Xing[5], Wenqing Li[1], Jiaqi Zou[1], Lan Meng[1], Muhammad Azhar[1], Yuzhu Cao[5], Xianhong Tong[5], Weibing Qin[6*], Xiaoli Zhu[5*], Jianqiang Bao[1,7*]

This combined PDF file contains the following Supplementary figures and tables:

Supplementary Figure 1. The breeding scheme for production of conditional Nat10 knockout mouse models.

Supplementary Figure 2. Nat10 is required for folliculogenesis.

Supplementary Figure 3. Nat10 KO caused disrupted transcriptome in GV oocytes.

Supplementary Figure 4. Nat10 KO elicited deficient maternal transcriptome in MII oocytes owing to aberrant transcript accumulation.

Supplementary Figure 5. Comparative study of three methods for examining poly(A) tail length (PAT) - HA-PAT, LM-PAT and ePAT in mouse oocytes.

Supplementary Figure 6. Validation of the aberrantly accumulated transcripts by LM-PAT in Nat10-ZcKO MII oocytes.

Supplementary Figure 7. Genome-wide profiling of translatome by Ribo-seq.

Supplementary Figure 8. Nat10 deposits ac4C modification on the key transcripts from CCR4-NOT complex genes.

Supplementary Figure 9. ac4C modification regulates the translation efficiency for transcripts enriched in translation, chromosome segregation and tRNA processing.

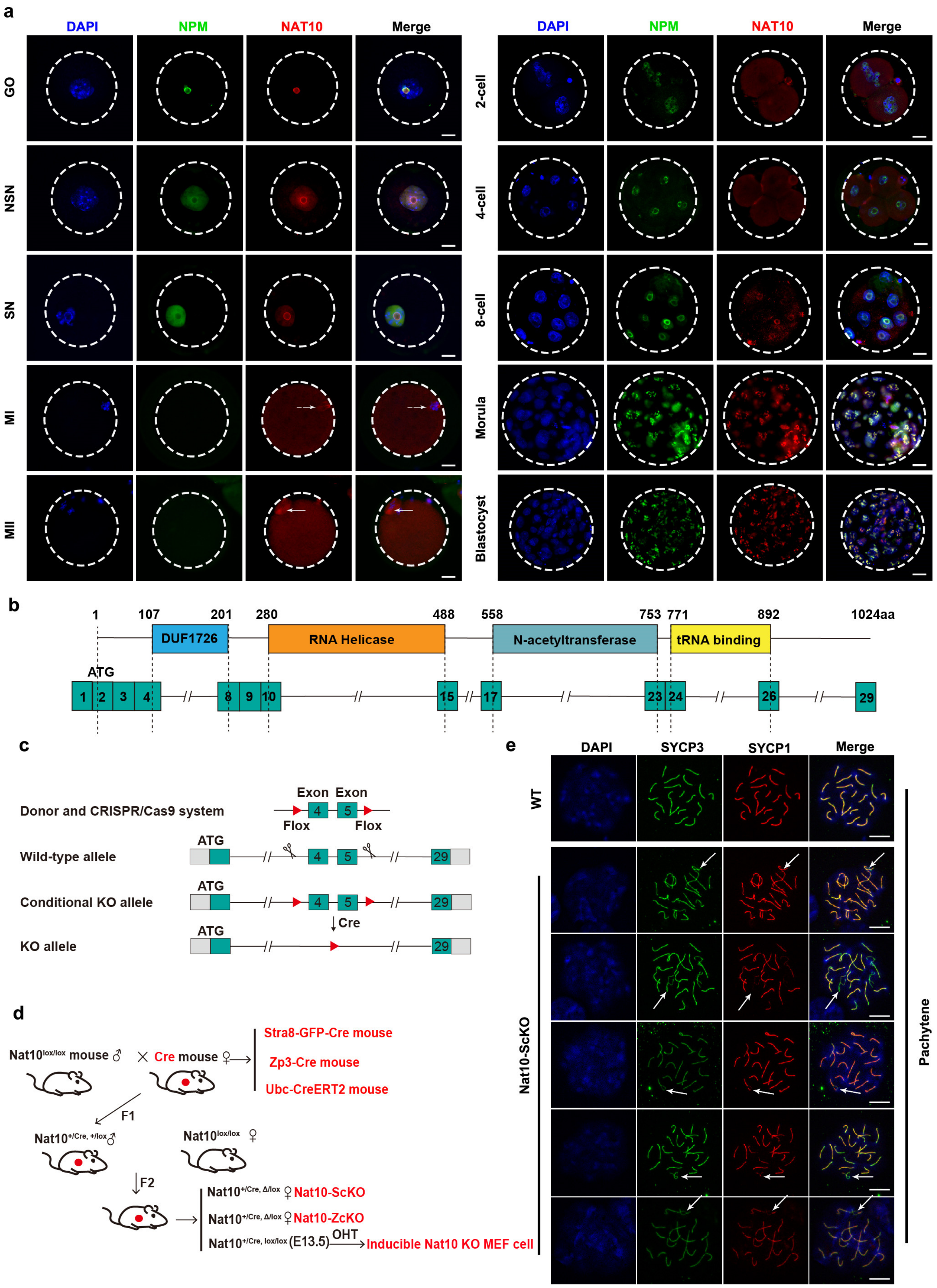

Supplementary Figure 1. The breeding scheme for production of conditional Nat10 knockout mouse models. a IF images of growing oocytes (GO), non-surrounded nucleolus (NSN), surrounded nucleolus (SN), MI, MII oocytes and 2-cell, 4-cell, 8-cell, morula and blastocyst by co-staining with NAT10 antibody (Red), Nucleophosmin (NPM, Green) and DAPI (Blue) as indicated. Dashed circle

indicates cellular membrane of oocytes or zona pellucida of embryos. Scale bar, 20 μm. **b** Schematic illustration displaying the conserved structural domains of mouse NAT10 protein. The floxed Exon 4 and 5 reside (E4/5) within DUF1726 domain. **c** Schematic representation of the Nat10lox/lox mouse allele. The crossing of floxed E4/5 ($Nat10^{lox/lox}$) with Cre deletor mice led to the E4/5 knockout, resulting in complete Nat10 knockout (*Nat10*-null) owing to frame-shift mutation. **d** Schematic diagram depicting the breeding strategy for the crossing of $Nat10^{lox/lox}$ allele with Cre deletor mice. **e** IF staining of SYCP3 and SYCP1 on surface-spread oocytes at pachytene stage from WT and Nat10-ScKO mice at birth. Scale bar, 10μm. Arrows point to the asynapsed structure between the lateral and central axes. n = 3 biologically independent samples were included in each group. Source data are provided as a source data file.

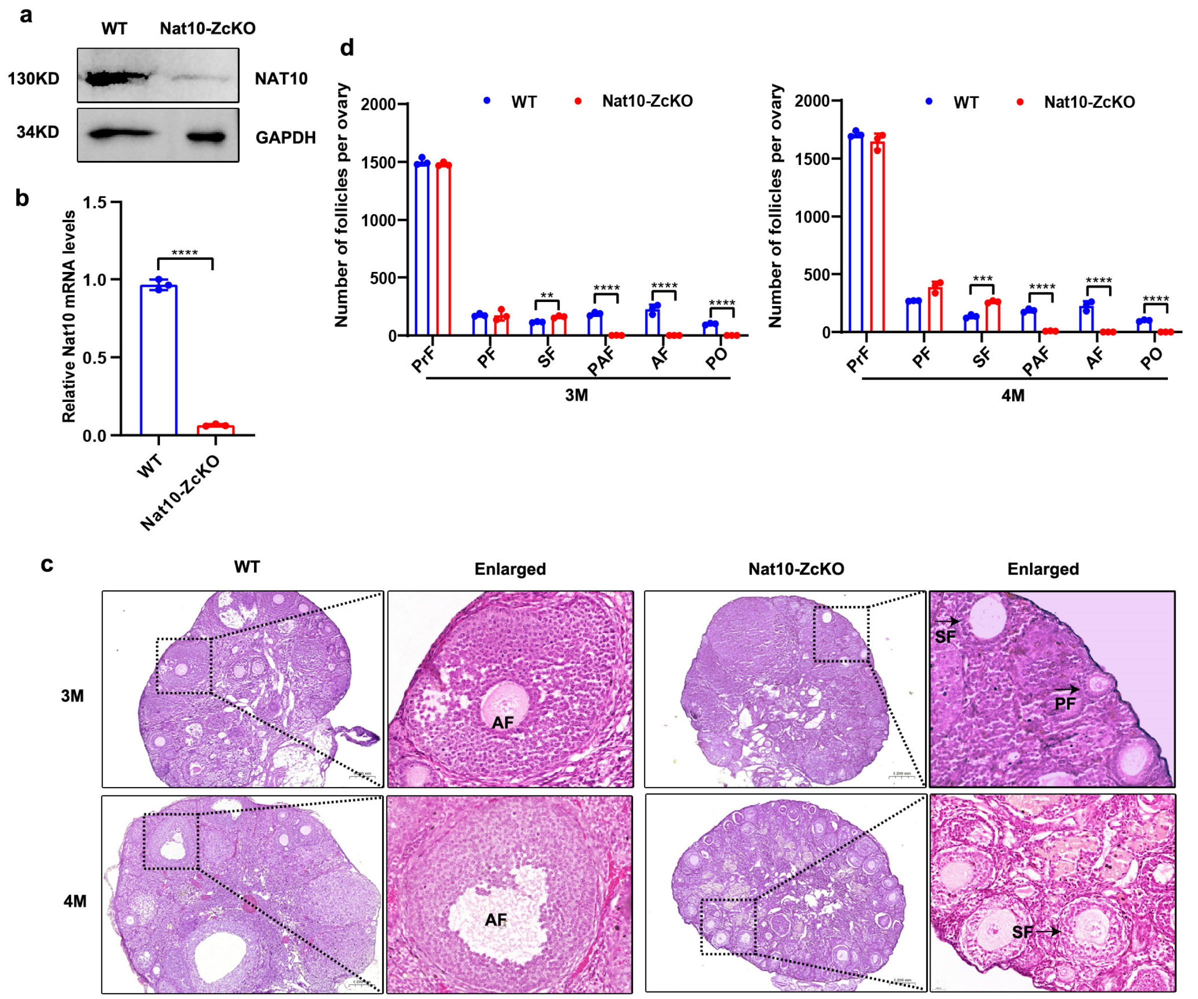

Supplementary Figure 2. Nat10 is required for folliculogenesis. a-b Oocyte-specific Nat10 knockout validation in the oocytes from Zp3-cre; Nat10lox/lox females by immunoblotting **a** and qRT-PCR quantification. Data are presented as the mean± SEM, n=3; ****, p<0.001 by two-tailed *Student's t-test*. **b**. **c** H&E staining showing ovarian histology of 3-month-old (Top), and 4-month-old (Bottom) mice with indicated genotypes. Scale bar, 200μm. Right panel is a magnified view of the dotted box in left panel. Follicles indicated by arrows. **d** Comparison of the average numbers of follicles at indicated stages in the ovaries of WT and Nat10-ZcKO mouse at 3-month-old (Left) and 4-month-old (Right). Follicles were counted on serial ovarian sections after H&E staining. Data are presented as the mean± SEM, n=3; **, p<0.01; ***, p<0.001; ****, p<0.0001 by two-tailed *Student's t-test*. 3M SF, p= 0.0021; 4M SF, p= 0.0003. n = 3 biologically independent samples were included in each group (a, c). Source data are provided as a source data file.

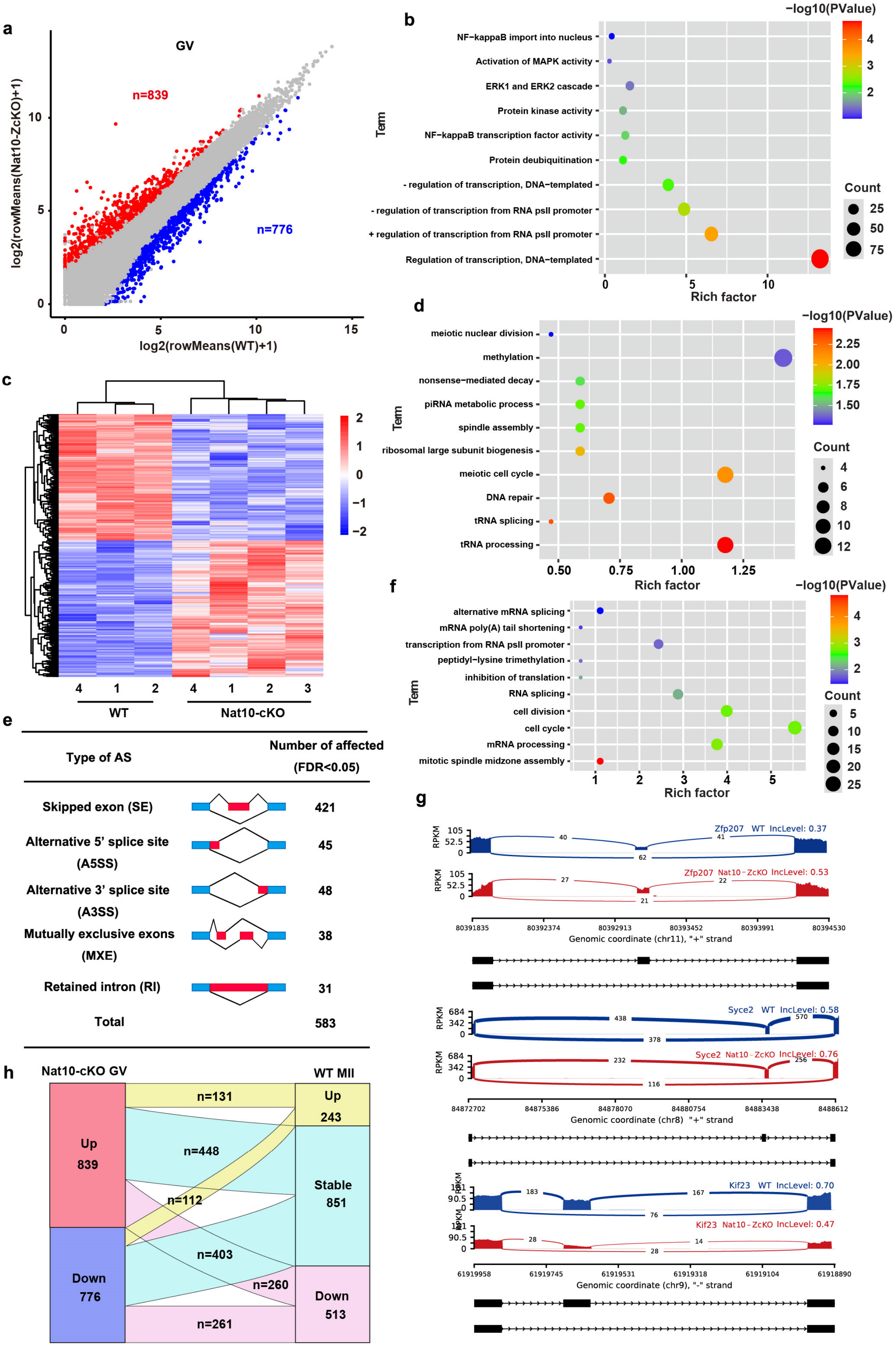
a
GV
n=839
n=776
log2(rowMeans(Nat10-ZcKO)+1)
log2(rowMeans(WT)+1)
b
−log10(PValue)
NF−kappaB import into nucleus
Activation of MAPK activity
ERK1 and ERK2 cascade
Protein kinase activity
NF−kappaB transcription factor activity
Protein deubiquitination
- regulation of transcription, DNA−templated
- regulation of transcription from RNA psII promoter
+ regulation of transcription from RNA psII promoter
Regulation of transcription, DNA−templated
Term
Rich factor
Count
c
WT
Nat10-cKO
d
meiotic nuclear division
methylation
nonsense−mediated decay
piRNA metabolic process
spindle assembly
ribosomal large subunit biogenesis
meiotic cell cycle
DNA repair
tRNA splicing
tRNA processing
e
Type of AS
Number of affected (FDR<0.05)
Skipped exon (SE) 421
Alternative 5' splice site (A5SS) 45
Alternative 3' splice site (A3SS) 48
Mutually exclusive exons (MXE) 38
Retained intron (RI) 31
Total 583
f
alternative mRNA splicing
mRNA poly(A) tail shortening
transcription from RNA psII promoter
peptidyl−lysine trimethylation
inhibition of translation
RNA splicing
cell division
cell cycle
mRNA processing
mitotic spindle midzone assembly
g
Zfp207 WT IncLevel: 0.37
Zfp207 Nat10−ZcKO IncLevel: 0.53
Genomic coordinate (chr11), "+" strand
Syce2 WT IncLevel: 0.58
Syce2 Nat10−ZcKO IncLevel: 0.76
Genomic coordinate (chr8) "+" strand
Kif23 WT IncLevel: 0.70
Kif23 Nat10−ZcKO IncLevel: 0.47
Genomic coordinate (chr9), "-" strand
RPKM
h
Nat10-cKO GV
WT MII
Up 839
Down 776
Up 243
Stable 851
Down 513
n=131
n=448
n=112
n=403
n=260
n=261

Supplementary Figure 3. Nat10 KO caused disrupted transcriptome in GV oocytes. a Scatter plot of mini-bulk SMART-seq2 data illustrating transcriptional changes in Nat10-ZcKO GV oocytes. Transcripts down-regulated or up-regulated in Nat10-ZcKO GV oocyte samples were highlighted in blue or red, respectively. (Cutoff: fold change (FC)$\geqslant$2, p<0.05). The TPMs of WT and Nat10-ZcKO GV oocytes are listed in Table S4. **b** GO enrichment analysis of biological processes (BP) for the down-regulated transcripts in the Nat10-ScKO GV oocytes. **c** Heatmap showing dysregulated transcripts in Nat10-ZcKO GV oocytes. **d** GO analysis for the up-regulated transcripts in Nat10-ZcKO GV oocytes. **e-g** Alternative splicing identification in the Nat10-ZcKO GV oocytes. A total of 583 alternative splicing events were detected in the Nat10-ZcKO GV oocytes **e**. GO analysis for the alternative spliced genes **f**. Representative alternative splicing genes as visualized by sashimi snapshot (rMATS) **g**. **h** Sankey plot showing the overlapping of the dysregulated genes in the Nat10-ZcKO GV oocytes with three types of maternal transcripts in the WT oocytes during GV-MII transition (Up, up-regulated transcripts during GV-MII transition; Stable, no change during GV-MII transition; Down, down-regulated transcripts during GV-MII transition). Source data are provided as a source data file.

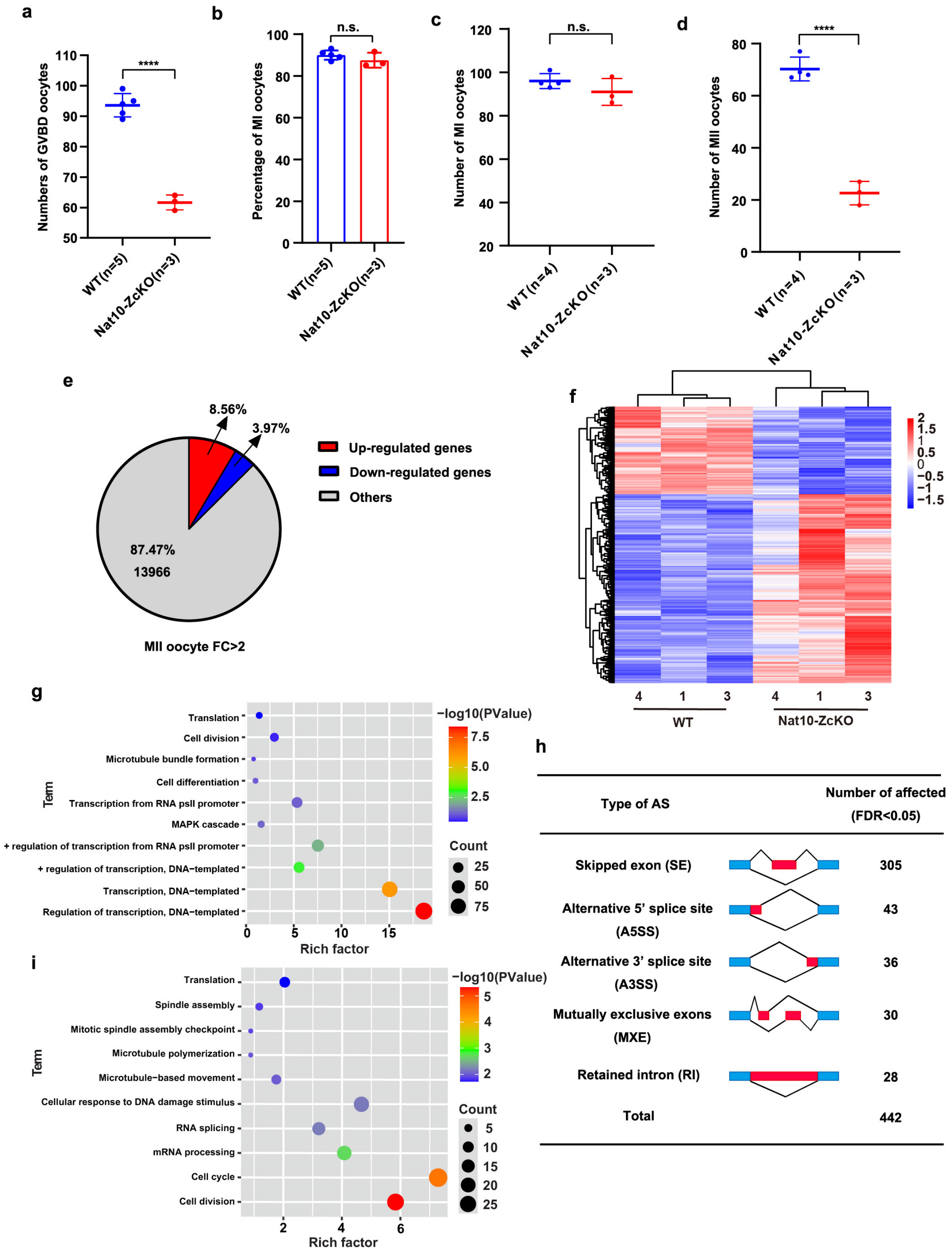

Supplementary Figure 4. Nat10 KO elicited deficient maternal transcriptome in MII oocytes owing to aberrant transcript accumulation. a-d Comparison of the average numbers of retrieved GV oocytes cultured in vitro that developed to GVBD **a**, MI **b-c** and MII **d** stages. Data are presented as the mean±SEM, n=3. ****, p<0.0001, n.s., non-significant by two-tailed *Student's t-test*. **e** Pie chart displaying the transcriptome-wide distribution of up-regulated genes (Red), down-regulated genes

(Blue) and the others (Gray) in Nat10-ZcKO MII oocytes. **f** Heatmap showing level changes in maternal transcripts in Nat10-ZcKO MII oocytes. **g** GO enrichment analysis of biological processes (BP) indicates the potential functions of the down-regulated transcripts in the Nat10-ZcKO MII oocytes (adjusted $p<0.05$, FC > 2). **h-i** Summary of the alternative splicing of transcripts in MII oocytes isolated from the WT and Nat10-ZcKO. The numbers of predicted alternative splicing (AS) events in each category upon Nat10 deletion are indicated **h**. GO enrichment analysis of biological processes (BP) indicates the potential functions of the splicing events affected by Nat10 KO in the MII oocytes **i**. Source data are provided as a source data file.

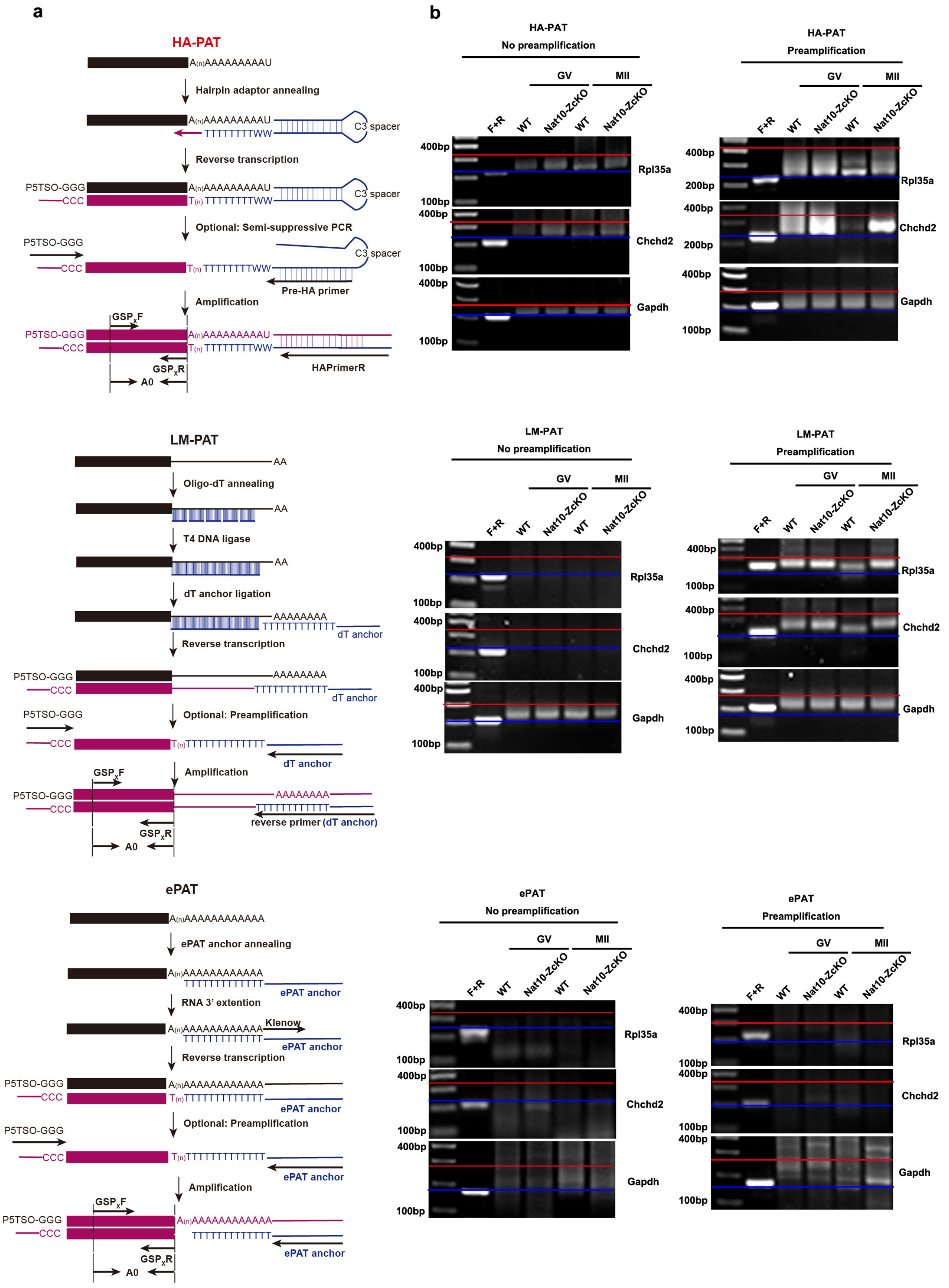

Supplementary Figure 5. Comparative study of three methods for examining poly(A) tail length (PAT) - HA-PAT, LM-PAT and ePAT in mouse oocytes. a Schematic diagram depicting the key steps executed in each method. Hairpin Adaptor PAT (HA-PAT) is a novel method developed in-house in this

study. GSP, Gene-specific primer. A0 indicates the PCR product that encompasses the full length amplified by GSP primers excluding the poly(A) tail length. The other two methods (Ligation-mediated PAT, LM-PAT and extension PAT, ePAT) were performed according to previous published procedures. HA-PAT requires less hands-on operations and reagents (Top panel). **b** Representative images showing the agarose gel electrophoresis of the amplified PCR products with or without preamplification in each approach using GV and MII oocytes from both WT and Nat10-ZcKO females. Aberrant accumulation of poly(A) mRNAs was visualized as stronger, and higher smear PCR bands as pointed out by blue and red lines. The in-house HA-PAT method is most sensitive as it gave rise to apparent signal intensity in oocytes without preamplification (Top panel). Upon preamplification, HA-PAT outperformed the other two methods in terms of sensitivity and signal intensity. Blue line points to lower boundary of smear PCR band. Red line points to the upper boundary of smear PCR band. n = 3 biologically independent samples were included in each group. Source data are provided as a source data file.

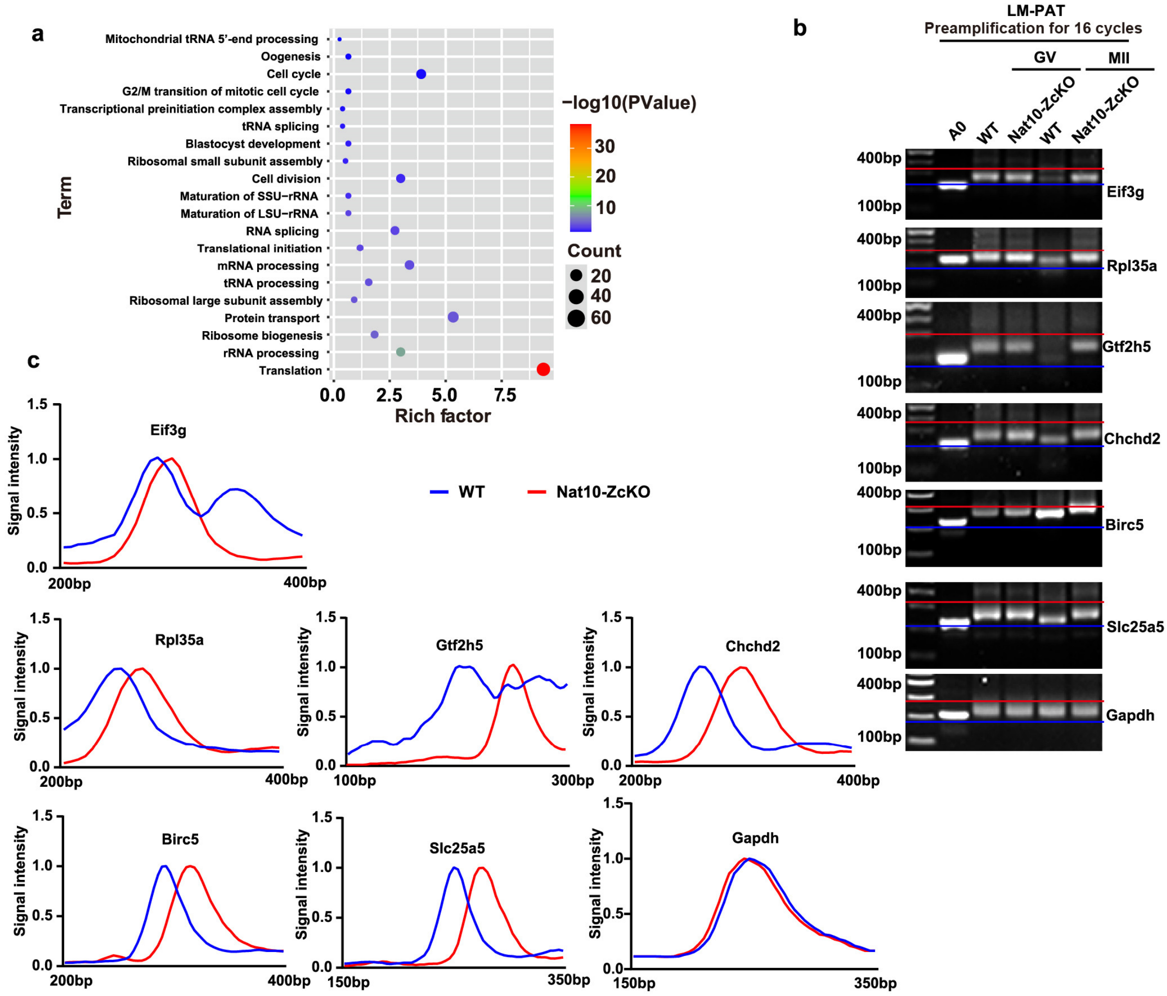

Supplementary Figure 6. Validation of the aberrantly accumulated transcripts by LM-PAT in Nat10-ZcKO MII oocytes. **a** GO enrichment analysis of the overlapping transcripts that were up-regulated in the MII oocytes derived from Nat10-ZcKO and Cnot6l$^{-/-}$ mice. **b** Representative images showing the agarose gel electrophoresis of the amplified PCR products by LM-PAT for a cohort of up-regulated genes as examined by HA-PAT in Figure 8. n = 3 biologically independent samples were included in each group. **c** The densitometric curves showing the poly(A) tail length distribution of the corresponding transcripts in **b**. n = 3 biologically independent samples were included in each group. Source data are provided as a source data file.

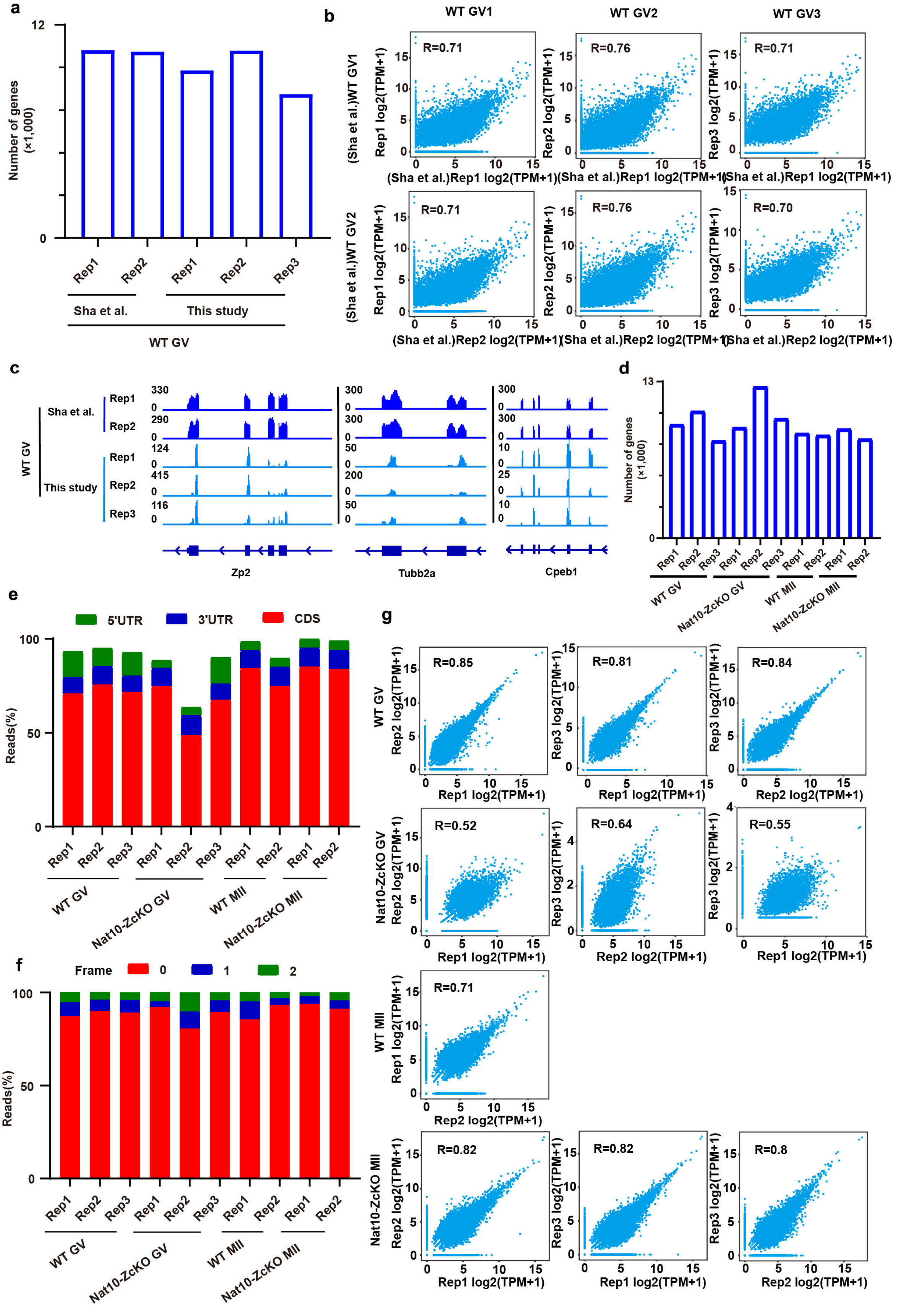

Supplementary Figure 7. Genome-wide profiling of translatome by Ribo-seq in the oocytes. a Bar plots showing the numbers of detected genes (TPM>1) by the published Ribo-seq data of Sha et

al. (two replicates, ‘Rep1’ and ‘Rep2’) and Ribo-seq in this study (three replicates, ‘Rep1’, ‘Rep2’ and ‘Rep3’). **b** Scatter plots comparing the Ribo-seq data from Sha et al. and this study. Spearman correlations are also shown. **c** The UCSC browser tracks of Ribo-seq signal (RPF) for Zp2, Tubb2a and Cpeb1. **d** Bar plots showing the numbers of genes detected by Ribo-seq (TPM > 1). **e** Bar plots showing the mapping reads distribution in 5’UTR, CDS and 3’UTR regions. **f** Bar plots showing the percentages of footprints that match the reading frames. **g** Scatter plots comparing replicates of Ribo-seq for GV and MII from WT and Nat10-ZcKO mice. Source data are provided as a source data file.

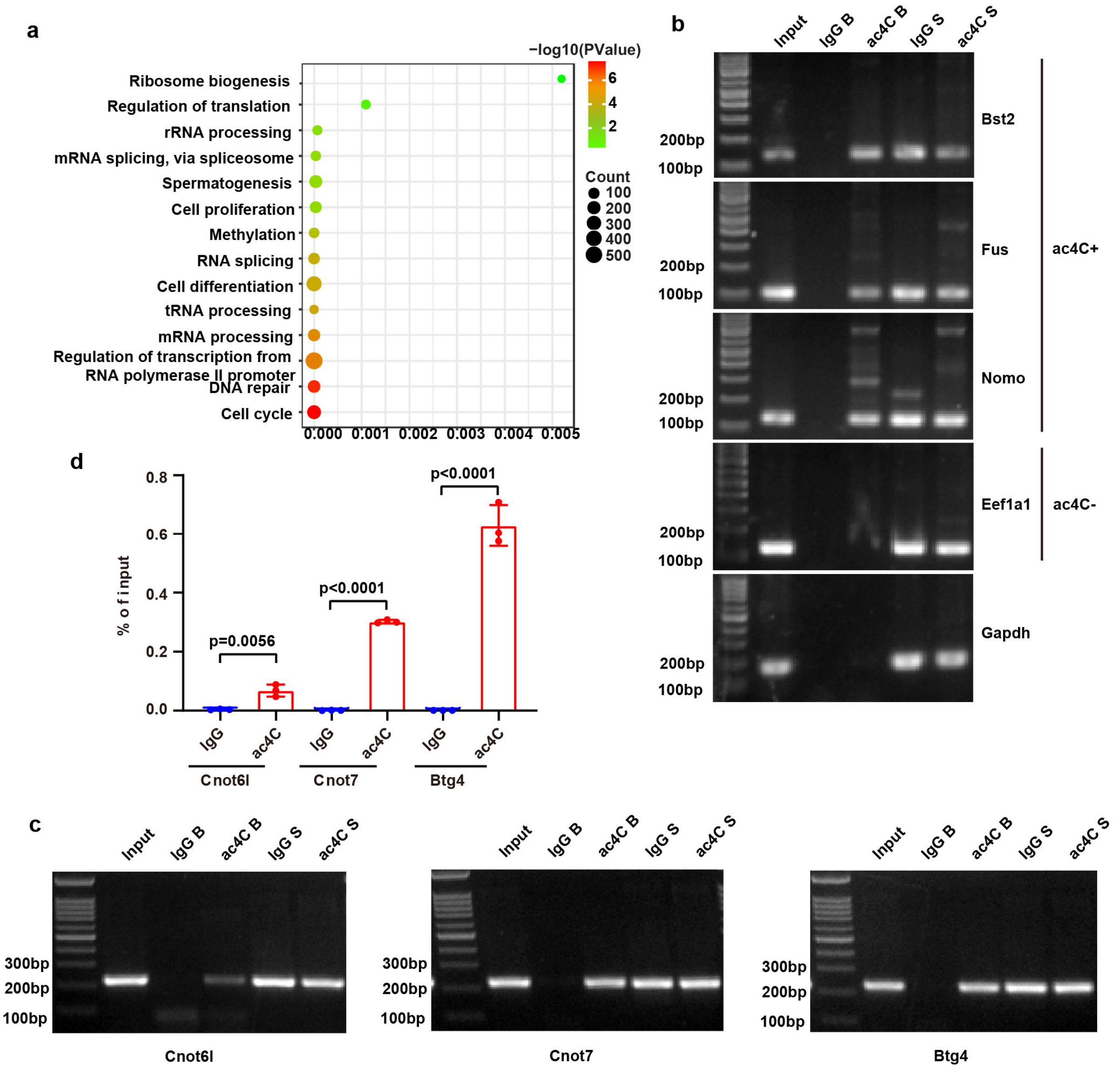

Supplementary Figure 8. Nat10 deposits ac4C modification on the key transcripts from CCR4-NOT complex genes. a Gene Ontology (GO) enrichment analysis of gene transcripts with ac4C modification identified by RIP-seq in the GV oocytes. **b** acRIP-PCR assay confirmed selected genes with or without ac4C modification in HeLa cells. IgG B, IgG beads. Ac4C B, ac4C beads. IgG S, IgG supernatant. Ac4C S, ac4C supernatant. Experiments were performed in triplicates; a representative image is shown in the 2% agarose gel. n = 3 biologically independent samples were included in each group. **c** acRIP-PCR results validated the ac4C modification on the transcripts for Cnot6l, Cnot7 and Btg4 in WT GV oocytes. n = 3 biologically independent samples were included in each group. **d** acRIP-qPCR assay showing the efficiency of IP for Cnot6l, Cnot7 and Btg4 transcripts in WT GV oocytes. Data are presented as the mean± SEM, n=3; ****, p<0.0001 by two-tailed *Student's t-test*. Source data are provided as a source data file.

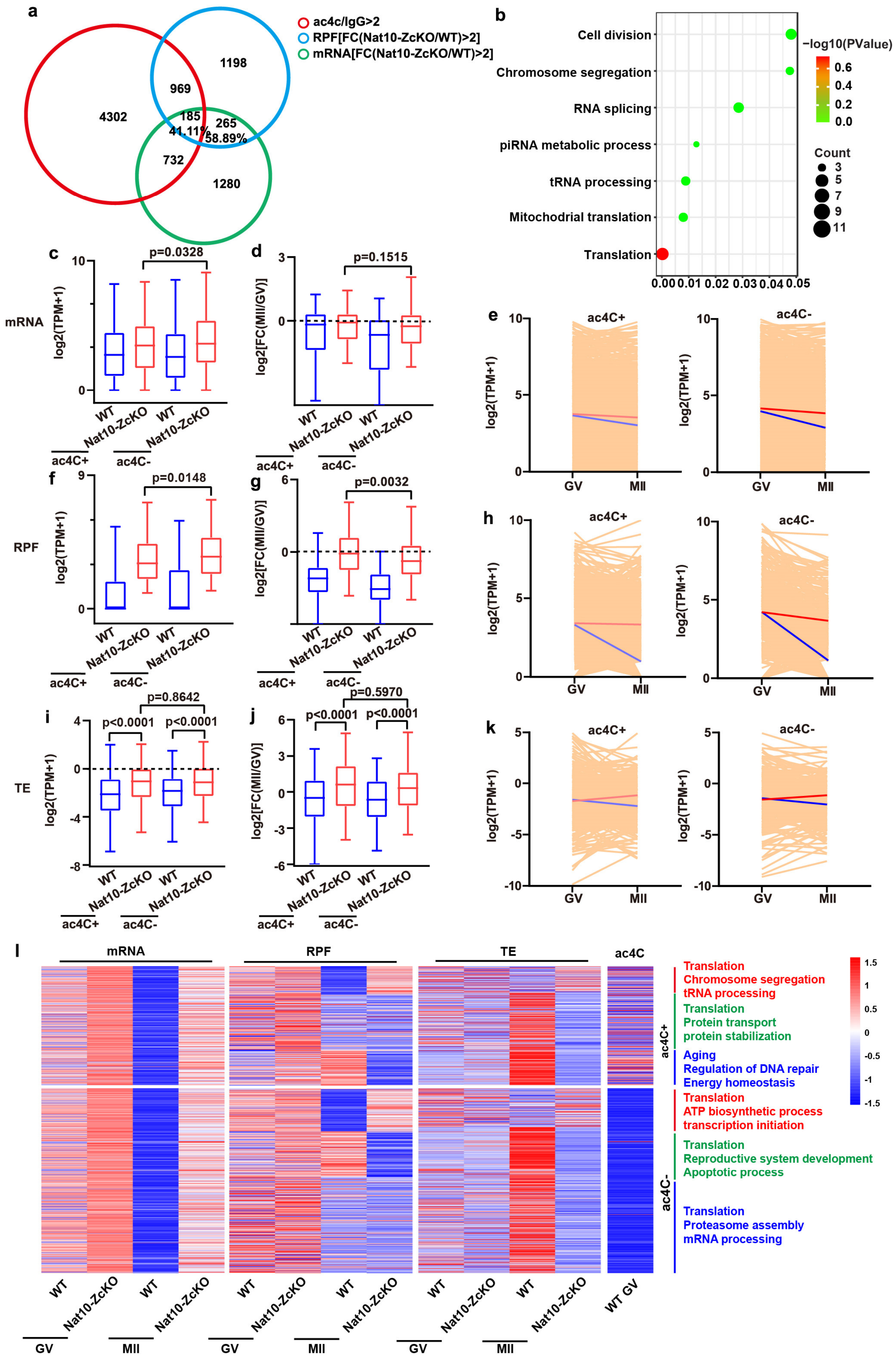
a
ac4c/IgG>2
RPF[FC(Nat10-ZcKO/WT)>2]
mRNA[FC(Nat10-ZcKO/WT)>2]
4302
969
1198
185
41.11%
265
58.89%
732
1280
b
Cell division
Chromosome segregation
RNA splicing
piRNA metabolic process
tRNA processing
Mitochondrial translation
Translation
0.00 0.01 0.02 0.03 0.04 0.05
−log10(PValue)
0.6
0.4
0.2
0.0
Count
3
5
7
9
11
c
mRNA
log2(TPM+1)
p=0.0328
d
log2[FC(MII/GV)]
p=0.1515
WT
Nat10-ZcKO
ac4C+
ac4C-
e
ac4C+
ac4C-
GV
MII
f
RPF
p=0.0148
g
p=0.0032
h
i
TE
p=0.8642
p<0.0001
j
p=0.5970
p<0.0001
k
l
mRNA
RPF
TE
ac4C
Translation
Chromosome segregation
tRNA processing
Translation
Protein transport
protein stabilization
Aging
Regulation of DNA repair
Energy homeostasis
Translation
ATP biosynthetic process
transcription initiation
Translation
Reproductive system development
Apoptotic process
Translation
Proteasome assembly
mRNA processing
1.5
1
0.5
0
-0.5
-1
-1.5
WT GV
GV
MII

Supplementary Figure 9. ac4C modification regulates the translation efficiency for transcripts enriched in translation, chromosome segregation and tRNA processing. a Venn diagram showing the overlapping of transcripts with ac4C modification (FC [TPM (ac4C/IgG)]>2), up-regulated RPF (FC [TPM (Nat10-ZcKO/WT)]>2) and up-regulated mRNA (FC[TPM(Nat10-ZcKO/WT)]>2). **b** Gene Ontology (GO) enrichment of overlapping genes in **a**. **c-d** Box plots showing the relative mRNA levels of 185 and 265 genes in WT and Nat10-ZcKO oocytes at the GV and MII stages as indicated. Data are presented as mean± SEM, n=3. P-value by two-tailed *Student's t-test*. The box indicates the upper and lower quantiles, the thick line in the box indicates the median and whiskers indicates 2.5th and 97.5th percentiles. **e** The trend patterns of mRNA with (185 genes) or without (265 genes) ac4C modification during the GV-MII transition. Each light-yellow line represents the levels of one RPF, and the blue and red lines represent the median expression levels in WT and Nat10-ZcKO, respectively. **f-g** Box plots showing the relative RPF levels of 185 and 265 genes in WT and Nat10-ZcKO oocytes at the GV and MII stages as indicated. The box indicates the upper and lower quantiles, the thick line in the box indicates the median and whiskers indicates 2.5th and 97.5th percentiles. Data are presented as mean± SEM, n=3 biologically independent samples. P-value by two-tailed *Student's t-test*. **h** The trend patterns of RPF with (185 genes) or without (265 genes) ac4C modification during the GV-MII transition in WT and Nat10-ZcKO oocytes as in **e**. **i-j** Box plots showing the relative TE levels of 185 and 265 genes in WT and Nat10-ZcKO oocytes at the GV and MII stages as indicated. The box indicates the upper and lower quantiles, the thick line in the box indicates the median and whiskers indicates 2.5th and 97.5th percentiles. Data are presented as mean± SEM, n=3 biologically independent samples. P-value by two-tailed *Student's t-test*. **k** The trend patterns of TE with (185 genes) or without (265 genes) ac4C modification during the GV-MII transition in WT and Nat10-ZcKO oocytes as in **e**. **l** Heatmap showing the integrative hierarchical clustering for all DEGs with the corresponding RPF levels, TE levels and ac4C levels. The enriched GO terms are listed. Source data are provided as a source data file.
