# Supplementary material for: Maternal NAT10 orchestrates oocyte meiotic cell-cycle progression and maturation in mice
Source: Nat Commun. 2023 Jun 22;14:3729. doi: 10.1038/s41467-023-39256-0 (PMC10287700; doi:10.1038/s41467-023-39256-0)
Supplement: Supplementary file 3 — Description of Additional Supplementary Files [file 41467_2023_39256_MOESM3_ESM.pdf]

Description of Additional Supplementary Files

File name: Supplementary Data 1
Description: Primer sequences for genotyping and Real-time PCR

File name: Supplementary Data 2
Description: Mini-bulk SMART-seq2, PAT, Ribo-seq and acRIP-seq

File name: Supplementary Data 3
Description: Quality control of RNA-seq results

File name: Supplementary Data 4
Description: DEGs between WT and Nat10-ZcKO GV oocytes

File name: Supplementary Data 5
Description: DEGs between WT and Nat10-ZcKO MII oocytes
