# Supplementary material for: Maternal NAT10 orchestrates oocyte meiotic cell-cycle progression and maturation in mice
Source: Nat Commun. 2023 Jun 22;14:3729. doi: 10.1038/s41467-023-39256-0 (PMC10287700; doi:10.1038/s41467-023-39256-0)
Supplement: Supplementary file 9 — Reporting Summary [file 41467_2023_39256_MOESM9_ESM.pdf]

nature portfolio

Corresponding author(s): Jianqiang Bao; Xiaoli Zhu; Weibing Qin

Last updated by author(s): May 13, 2023

Reporting Summary

Nature Portfolio wishes to improve the reproducibility of the work that we publish. This form provides structure for consistency and transparency in reporting. For further information on Nature Portfolio policies, see our Editorial Policies and the Editorial Policy Checklist.

Statistics

For all statistical analyses, confirm that the following items are present in the figure legend, table legend, main text, or Methods section.

n/a	Confirmed	
☐	☒	The exact sample size (*n*) for each experimental group/condition, given as a discrete number and unit of measurement
☐	☒	A statement on whether measurements were taken from distinct samples or whether the same sample was measured repeatedly
☐	☒	The statistical test(s) used AND whether they are one- or two-sided<br>*Only common tests should be described solely by name; describe more complex techniques in the Methods section.*
☐	☒	A description of all covariates tested
☐	☒	A description of any assumptions or corrections, such as tests of normality and adjustment for multiple comparisons
☐	☒	A full description of the statistical parameters including central tendency (e.g. means) or other basic estimates (e.g. regression coefficient) AND variation (e.g. standard deviation) or associated estimates of uncertainty (e.g. confidence intervals)
☒	☐	For null hypothesis testing, the test statistic (e.g. *F*, *t*, *r*) with confidence intervals, effect sizes, degrees of freedom and *P* value noted<br>*Give P values as exact values whenever suitable.*
☒	☐	For Bayesian analysis, information on the choice of priors and Markov chain Monte Carlo settings
☒	☐	For hierarchical and complex designs, identification of the appropriate level for tests and full reporting of outcomes
☒	☐	Estimates of effect sizes (e.g. Cohen's *d*, Pearson's *r*), indicating how they were calculated

Our web collection on statistics for biologists contains articles on many of the points above.

Software and code

Policy information about availability of computer code

Data collection	NanoPhotometer N50; Q2000B OPTIMAL; Mshot; SHST; Leica Application Suite X; ISCO fractionator; NovaSeq 6000 platform
Data analysis	R software; STAR; RSEM; rMATs; DAVID; DEseq2; Image J; FlowJo V10 software, Western blot densitometry was determined using the Histogram function of Adobe Photoshop 2020 version 21.1.2. qRT-PCR data was analyzed in Microsoft Excel version 16.36. Graphs were plotted using GraphPad Prism 8 version 8.4.2. Statistical analysis was done in GraphPad Prism 8

For manuscripts utilizing custom algorithms or software that are central to the research but not yet described in published literature, software must be made available to editors and reviewers. We strongly encourage code deposition in a community repository (e.g. GitHub). See the Nature Portfolio guidelines for submitting code & software for further information.

Data

Policy information about availability of data

All manuscripts must include a data availability statement. This statement should provide the following information, where applicable:

- Accession codes, unique identifiers, or web links for publicly available datasets
- A description of any restrictions on data availability
- For clinical datasets or third party data, please ensure that the statement adheres to our policy

All the databases used in the study along with appropriately accessible links in the manuscript under the "Data availability" section. All the raw data and processed files have been deposited in the Gene Expression Omnibus (http://www.ncbi.nlm.nih. gov/geo) with the accession number: SRP392832 and GSE228721. The source

data were provided as a source data file. and protocols that support the findings of this study will be made available by corresponding author upon reasonable request

Human research participants

Policy information about studies involving human research participants and Sex and Gender in Research.

Reporting on sex and gender	None
Population characteristics	None
Recruitment	None
Ethics oversight	None

Note that full information on the approval of the study protocol must also be provided in the manuscript.

Field-specific reporting

Please select the one below that is the best fit for your research. If you are not sure, read the appropriate sections before making your selection.

☒ Life sciences ☐ Behavioural & social sciences ☐ Ecological, evolutionary & environmental sciences

For a reference copy of the document with all sections, see nature.com/documents/nr-reporting-summary-flat.pdf

Life sciences study design

All studies must disclose on these points even when the disclosure is negative.

Sample size	For each phenotypic experiment, we needed five WT and five KO mice to satisfy three biological replicates. For Smart-seq2, we optimized the sequencing method that only five oocytes were needed for its initial quantity, so three mice were needed for WT and three mice for KO. five oocytes were enough to do smart-seq2, because previous study used one/ five cell/ oocytes to do this experiment. For Ribo-Seq, we uesd 100 200 and 500 GV oocytes to test our experiment, and three initial measurements measured high reproducibility of the data. And MII oocytes of Nat10-ZcKO mice were so difficult to get 500 oocytes. We needed 100 oocytes per replicate, for a total of fifteen WT and forty KO mice. For RIP-seq, We refer to previous published work based on m6A RIP-seq, that were used 1000 oocytes. But compared with the modification level of m6A on mRNA, ac4C is so low, so we need 2,000 oocytes for ac4C RIP-seq. The whole article cost was about 100 WT mice and 200 KO mice.
Data exclusions	None data were excluded
Replication	At least three biological replicates for each experiment. RIP-seq was performed in a pool of 2000 GV oocytes from 20 mice.
Randomization	Random distribution
Blinding	yes

Reporting for specific materials, systems and methods

We require information from authors about some types of materials, experimental systems and methods used in many studies. Here, indicate whether each material, system or method listed is relevant to your study. If you are not sure if a list item applies to your research, read the appropriate section before selecting a response.

Materials & experimental systems

n/a	Involved in the study	
☐	☒	Antibodies
☐	☒	Eukaryotic cell lines
☒	☐	Palaeontology and archaeology
☐	☒	Animals and other organisms
☒	☐	Clinical data
☒	☐	Dual use research of concern

Methods

n/a	Involved in the study	
☒	☐	ChIP-seq
☒	☐	Flow cytometry
☒	☐	MRI-based neuroimaging

Antibodies

Antibodies used

rabbit anti-NAT10 (ZENBIO, 389412, 1:1000), mouse anti-PCNA (Proteintech, 60097-1-Ig, 1:1000), mouse anti-GAPDH (Proteintech, 60004-1-Ig, 1:5000), rabbit anti-Tubulin (Proteintech, 11224-1-AP,1:5000). rabbit anti-CNOT6/6L (Abcam, ab86209,1:1000), rabbit anti-BTG4 (Abcam, ab235085,1:1000),mouse anti-SYCP3 (Abcam, ab97672,1:1000), rabbit anti-SYCP1 (Abcam, ab15090,1:1000), rabbit anti-SYCP3 (Proteintech, 23024-1-AP,1:200), mouse anti-γH2AX (Millipore, 05-636, 1:1000), rabbit anti-RPA2 (Proteintech, 10412-1-AP, 1:400), TRITC-conjugated Goat Anti-Rabbit IgG (Proteintech, SA00007-2, 1:500), 488-conjugated secondary antibodies (Proteintech, gb2AF488, 1:500),rabbit anti-NAT10 (Proteintech, 13365-1-AP,1:400), rabbit anti-Nucleophosmin (Abcam, ab10530, 1:1000), rabbit anti-H3K4me3 (Abclonal, A2357, 1:200), rabbit anti-α-Tubulin (Proteintech, 11224-1-AP, 1:200), rabbit anti-KI67 (Servicebio, GB111141, 1:400), anti-ac4C polyclonal antibody (Abcam, ab252215, 1:200) or anti-IgG polyclonal antibody (Proteintech, 30000-0-AP, 1:1000)

Validation

rabbit anti-NAT10 (ZENBIO, 389412) was used to detect the expression level by WB. Species Reactivity: Human, Mouse, Rat. Applications: WB, IHC, IF, Flow Cytometry.
mouse anti-PCNA (Proteintech, 60097-1-Ig) was previously used (PKM2 allosteric converter: A self-assembly peptide for suppressing renal cell carcinoma and sensitizing chemotherapy; Liver-specific deficiency of unc-51 like kinase 1 and 2 protects mice from acetaminophen-induced liver injury.)
mouse anti-GAPDH (Proteintech, 60004-1-Ig) was previously used (A signalling pathway for transcriptional regulation of sleep amount in mice)
rabbit anti-Tubulin (Proteintech, 11224-1-AP) was previously used (Regulation of m6A Transcripts by the 3'→5' RNA Helicase YTHDC2 Is Essential for a Successful Meiotic Program in the Mammalian Germline)
rabbit anti-CNOT6/6L (Abcam, ab86209) was previously used (CNOT6L couples the selective degradation of maternal transcripts to meiotic cell cycle progression in mouse oocyte)
rabbit anti-BTG4 (Abcam, ab235085) was previously used (Homozygous Mutations in BTG4 Cause Zygotic Cleavage Failure and Female Infertility)
mouse anti-SYCP3 (Abcam, ab97672) was previously used (The Spin1 interactor, Spindoc, is dispensable for meiotic division, but essential for haploid spermatid development in mice)
rabbit anti-SYCP1 (Abcam, ab15090) was previously used (The Spin1 interactor, Spindoc, is dispensable for meiotic division, but essential for haploid spermatid development in mice)
rabbit anti-SYCP3 (Proteintech, 23024-1-AP) was previously used (YTHDC2 is essential for pachytene progression and prevents aberrant microtubule-driven telomere clustering in male meiosis)
mouse anti-γH2AX (Millipore, 05-636) was previously used (Mouse BAZ1A (ACF1) is dispensable for double-strand break repair but is essential for averting improper gene expression during spermatogenesis)
rabbit anti-RPA2 (Proteintech, 10412-1-AP) was previously used (The ZFP541-KCTD19 complex is essential for pachytene progression by activating meiotic genes during mouse spermatogenesis)
TRITC-conjugated Goat Anti-Rabbit IgG (Proteintech, SA00007-2) was previously used (Newly synthesized AIFM1 determines the hypersensitivity of T lymphocytes to STING activation-induced cell apoptosis)
488-conjugated secondary antibodies (Proteintech, gb2AF488) was previously used (Three-dimensional structured illumination microscopy with enhanced axial resolution)
rabbit anti-NAT10 (Proteintech, 13365-1-AP) was previously used (NAT10-mediated N4-acetylcytidine modification is required for meiosis entry and progression in male germ cells)
rabbit anti-Nucleophosmin (Abcam, ab10530) was previously used (Nucleolar localization of the ErbB3 receptor as a new target in glioblastoma)
rabbit anti-H3K4me3 (Abclonal, A2357) was previously used (The Nuclear Matrix Protein SAFB Cooperates with Major Satellite RNAs to Stabilize Heterochromatin Architecture Partially through Phase Separation)
rabbit anti-α-Tubulin (Proteintech, 11224-1-AP) was previously used (Cilia locally synthesize proteins to sustain their ultrastructure and functions)
rabbit anti-KI67 (Servicebio, GB111141) was previously used (The transcription factor Sox7 modulates endocardiac cushion formation contributed to atrioventricular septal defect through Wnt4/Bmp2 signaling)
anti-ac4C polyclonal antibody (Abcam, ab252215) was previously used (NAT10-mediated N4-acetylcytidine modification is required for meiosis entry and progression in male germ cells)
anti-IgG polyclonal antibody (Proteintech, 30000-0-AP) was previously used (AQP4 Aggravates Cognitive Impairment in Sepsis-Associated Encephalopathy through Inhibiting Nav 1.6-Mediated Astrocyte Autophagy)

Eukaryotic cell lines

Policy information about cell lines and Sex and Gender in Research

Cell line source(s)

MEF cells from embryos of C57BL/6J mice at E13.5 following a standard 3T3 protocol (Preparation, Culture, and Immortalization UNIT 28.1 of Mouse Embryonic Fibroblasts), 293T cells from ATCC

Authentication

STR analysis was performed on 293T cell lines. Standard isolation techniques were used to generate MEFs from day E13.5 embryos and primary melanocytes from the skin of neonatal C57BL/6J mice. Formal authentication was not performed on these cells. We generated two tamoxifen-inducible, stable cell lines following a standard 3T3 protocol. The genotype of all MEF cell lines was verified by PCR and/or WB.

Mycoplasma contamination

DAPI immunofluorescence staining was performed on the MEF cells and 293T cells, and no mycoplasma nuclear staining was found around the cells

Commonly misidentified lines
(See ICLAC register)

no

Animals and other research organisms

Policy information about studies involving animals; ARRIVE guidelines recommended for reporting animal research, and Sex and Gender in Research

Laboratory animals	All mice(WT, Nat10 lox/lox, Nat10-ScKO and Nat10-ZcKO) (1-day, 5-day, 7-day ,12-day, 15-day, 21-day, 1 Month, 2 Months, 3 Months and 4 Months) were from the C57BL/6J background, and were bred in a specific pathogen-free (SPF) facility with a 12h light/dark cycle and with free access to food and water. All animal experiments were approved by the Animal Care and Research Committee of the University of Science and Technology of China (USTC). We did phenotype and sequencing by using female mice. The mice was sacrificed by Cervical dislocation for the oocytes.
Wild animals	None
Reporting on sex	We found that Nat10-ScKO male mice were sterile and had smaller testis. However, we found that the causes of sterility in male mice are not the same as those in female mice, so we separately studied the mechanism of Nat10 loss on sterility in male and female mice
Field-collected samples	none
Ethics oversight	All animal experiments were approved by the Animal Care and Research Committee of the University of Science and Technology of China (USTC).

Note that full information on the approval of the study protocol must also be provided in the manuscript.
